# Exposure profiles of social-environmental neighborhood factors and persistent distressing psychotic-like experiences across four years among young adolescents in the US

**DOI:** 10.1017/S0033291725000224

**Published:** 2025-02-17

**Authors:** Benson S. Ku, Qingyue E. Yuan, Grace Christensen, Lina V. Dimitrov, Benjamin Risk, Anke Huels

**Affiliations:** 1 Department of Psychiatry and Behavioral Sciences, Emory University School of Medicine, Atlanta, GA, USA; 2 Department of Epidemiology, Rollins School of Public Health, Emory University, Atlanta, GA, USA; 3 Department of Biostatistics and Bioinformatics, Rollins School of Public Health, Emory University, Atlanta, GA, USA; 4 Gangarosa Department of Environmental Health, Rollins School of Public Health, Emory University, Atlanta, GA, USA

**Keywords:** neighborhood characteristics, physical activities, psychotic-like experiences, social determinants of health, team sports

## Abstract

**Background:**

Recent research has demonstrated that domains of social determinants of health (SDOH) (e.g. air pollution and social context) are associated with psychosis. However, SDOHs have often been studied in isolation. This study investigated distinct exposure profiles, estimated their associations with persistent distressing psychotic-like experiences (PLE), and evaluated whether involvement in physical activity partially explains this association.

**Methods:**

Analyses included 8,145 young adolescents from the Adolescent Brain and Cognitive Development Study. Data from the baseline and three follow-ups were included. Area-level geocoded variables spanning various domains of SDOH, including socioeconomic status, education, crime, built environment, social context, and crime, were clustered using a self-organizing map method to identify exposure profiles. Generalized linear mixed modeling tested the association between exposure profiles and persistent distressing PLE and physical activities (i.e. team and individual sports), adjusting for individual-level covariates including age, sex, race/ethnicity, highest level of parent education, family-relatedness, and study sites.

**Results:**

Five exposure profiles were identified. Compared to the reference Profile 1 (suburban affluent areas), Profile 3 (rural areas with low walkability and high ozone), and Profile 4 (urban areas with high SES deprivation, high crime, and high pollution) were associated with greater persistent distressing PLE. Team sports mediated 6.14% of the association for Profile 3.

**Conclusions:**

This study found that neighborhoods characterized by rural areas with low walkability and urban areas with high socioeconomic deprivation, pollution concentrations, and crime were associated with persistent distressing PLE. Findings suggest that various social-environmental factors may differentially impact the development of psychosis.

## Introduction

Psychotic-like experiences (PLEs), also referred to as subclinical psychotic symptoms or psychotic experiences, are odd or unreal perceptions, thoughts, or beliefs (Hinterbuchinger & Mossaheb, 2021). It is one of the earliest manifestations of psychotic disorders and can be common among children (Linscott & van Os, 2013; Schultze-Lutter et al., 2014). PLEs in childhood are associated with elevated rates of schizophrenia diagnoses and suicide attempts in adulthood (Fisher et al., 2013; Poulton et al., 2000). Additionally, persistent and distressing PLE may be more indicative of psychopathology later on in life with greater functional impairment, greater cognitive impairment, and increased mental health service utilization (Karcher et al., 2022; Oh et al., 2024). Given the high prevalence of PLE in early adolescence and its clinical significance to later risk for psychosis and general psychopathology, exploring the risk factors and mechanisms underlying persistent and distressful PLE is crucial to improve psychiatric risk assessments and more effective prevention strategies in children and adolescents.

It has long been known that urban upbringing is a major risk factor for developing psychotic disorders, including schizophrenia (March et al., 2008; Pignon, Szoke, Ku, Melchior, & Schurhoff, 2023). Under the social determinants of health model (SDOH) for psychosis, certain environmental and social conditions (e.g. pollution concentrations, social fragmentation, and socioeconomic deprivation) may impact downstream psychosocial and biological processes and, in turn, lead to psychosis (B. S. Ku, Addington, et al., 2023; D. M. Anglin et al., 2021; Karcher, Schiffman, & Barch, 2021; Walker et al., 2024). Early exposure to air pollution has been associated with increased odds of psychotic-like experiences in adolescence, potentially mediated by inflammation and excessive oxidative stress (Newbury et al., 2024). The Social Disorganization Theory posits that the wider community plays an important role in shaping social interactions, whether through sports or extracurricular activities, which may be crucial for mental health, especially for children (Ku, Compton, Walker, & Druss, 2021). For example, youth living in neighborhoods that were more walkable, had less crime, and greater social cohesion participated in more physical activities, particularly team sports (Wang, Narcisse, & McElfish, 2023; Xiao et al., 2023). And greater involvement with team sports, as opposed to individual sports, was shown to be associated with reduced risk for future psychopathology, including persistent distressing PLE (Hoffmann, Barnes, Tremblay, & Guerrero, 2022; B. S. Ku, Q. Yuan, et al., 2024). Perhaps structural factors may facilitate youth coming together to play team sports, and certain aspects of these social ties may be relevant to offset the risk of psychosis (B. S. Ku, Q. Yuan, et al., 2024).

Prior literature has largely focused on analyzing each neighborhood characteristic as it relates to psychosis separately (B. S. Ku, Q. Yuan, et al., 2024; Deidre M. Anglin et al., 2023; Ku, Addington, et al., 2021; B. S. Ku, J. Ren, et al., 2024; O’Donoghue, Roche, & Lane, 2016). Existing measurement tools, such as the Area Deprivation Index (ADI) and Child Opportunity Index (COI), have been limited in their ability to capture the multifaceted impacts of various neighborhood characteristics simultaneously while also highlighting individual contributions of each neighborhood characteristic (Benson S. Ku et al., 2024; Sandel et al., 2016). These indices are constructed from a chosen set of SDOH variables, and little is currently known about the combinations of these environmental and social factors as well as the mechanisms through which they lead to psychosis. Without such information, it may be difficult to devise effective, targeted policies and interventions.

This study aimed to fill this knowledge gap by using innovative methodologies that capture and analyze SDOH’s multidimensional nature and uncover their association with distressing PLE and physical activities. We hypothesized that certain exposure profiles of neighborhood characteristics would be associated with persistent distressing PLE and that team sports would partially explain this association.

## Methods

The data were collected from a population-based sample of 9-to-10-year-olds in the Adolescent Brain and Cognitive Development Study (ABCD) 5.0 release, which included visits collected between 1st, September 2016, and 15th, January 2022 (Fan et al., 2021). Data from the baseline and three follow-up time points were included.

The ABCD Study is a nationwide longitudinal study of brain, behavioral, and cognitive development in adolescents and involves 22 research sites throughout the United States, with more than 11,868 children recruited at baseline. Within these sites, public, private, and public charter schools within a 50-mile radius of the data-collecting site were randomly selected for recruitment (Garavan et al., 2018). Children aged 9–10 years old enrolled in selected primary schools were included for recruitment. Those with diagnoses of schizophrenia, autism spectrum disorder, substance use disorder, or medical or intellectual disability were excluded from the study (ABCD Study Team, 2024). Informed written consent was obtained from their parents and assent from children, and ethical approval was obtained from each site’s institutional review boards. We followed the Strengthening the Reporting of Observational Studies in Epidemiology (STROBE) reporting guidelines.

### Participants

This study included 8,145 participants based on the availability of environmental exposures, outcome variables, and sociodemographic covariates. Supplementary efigure 1 shows a flowchart of included and excluded participants. Supplementary eTable 1 compares those included and excluded based on missing data.

### Sociodemographic and clinical characteristics

Sociodemographic characteristics, including age, sex, race, ethnicity, parental education, household income, and family history of psychosis, were collected through parent reports and interviews during baseline assessment. Race and ethnicity were aggregated into five categories (non-Hispanic White, non-Hispanic Black, non-Hispanic Asian, non-Hispanic other races, and Hispanic), a schema commonly used in the ABCD study (Saragosa-Harris et al., 2022). High parental education was defined as having at least one parent or caregiver who obtained a bachelor’s degree or higher. The income-to-needs ratio was calculated by the median value of the income band divided by the federal poverty line for the respective household size (Rakesh, Zalesky, & Whittle, 2022). A value greater or less than one would denote above or below the poverty threshold, respectively. Family history of psychosis in first-degree and second-degree relatives was assessed using the parent-rated Family History Assessment Module Screener (Van Dijk, Murphy, Posner, Talati, & Weissman, 2021).

### Area-level exposures

Area-level data were derived from participants’ primary home addresses at baseline, which were geocoded to retrieve information at the census tract level. These geospatial location data were then linked to external environmental constructs, which were part of the ABCD 5.0 release (Fan et al., 2021). Exposure variables were used to construct five domains, including the Area Deprivation Index (ADI) (Kind et al., 2014), Child Opportunity Index 2.0 (COI) (Acevedo-Garcia et al., 2014), Crime (Investigation, 2014), Environmental Quality (Requia et al., 2020), and Social Vulnerability Index (SVI) (Fatemi, Ardalan, Aguirre, Mansouri, & Mohammadfam, 2017) based on their respective sources and prior literature (Fan et al., 2021). A comprehensive list of variables included in our study, exposure definition, and years measured are described in Table 1 and Supplementary eTable 2. Distance to major roadways, population density, and percentage of households without a car, were used to classify the exposure profiles as urban, suburban, and rural as done in prior literature (Ostermeijer, Koster, Van Ommeren, & Nielsen, 2022).

**Table 1.** Description of study sample by exposure profiles. A. Study sample characteristics. B. Area-level deprivation index. C. Child opportunity index. D. Crime. E. Environmental quality. F. Social vulnerability index. G. Urbanicity features. H. Physical activities. I. Outcome variable^a^

		**Exposure profiles**					
**Variables**	**Total**	**1**	**2**	**3**	**4**	**5**	**P**^b^
**A. Sample characteristics**							
N (%)	8,145 (100%)	2,521 (30.9%)	2,670 (32.8%)	1,459 (17.9%)	715 (8.8%)	780 (9.6%)	
Age (mean (SD))	9.92 (0.63)	9.95 (0.63)	9.93 (0.63)	9.90 (0.63)	9.89 (0.63)	9.87 (0.61)	0.006
Female Sex (%)	3,868 (47.5)	1,152 (45.7)	1,281 (48.0)	717 (49.1)	349 (48.8)	369 (47.3)	0.230
Race/ethnicity (%)							<.001
Non-Hispanic White	4711 (57.8)	1866 (74.0)	1675 (62.7)	873 (59.8)	123 (17.2)	174 (22.3)	
Non-Hispanic Black	912 (11.2)	64 (2.5)	250 (9.4)	133 (9.1)	65 (9.1)	400 (51.3)	
Non-Hispanic Asian	169 (2.1)	81 (3.2)	61 (2.3)	12 (0.8)	13 (1.8)	2 (0.3)	
Non-Hispanic others^c^	873 (10.7)	278 (11.0)	324 (12.1)	121 (8.3)	50 (7.0)	100 (12.8)	
Hispanic ethnicity	1480 (18.2)	232 (9.2)	360 (13.5)	320 (21.9)	464 (64.9)	104 (13.3)	
Parent bachelor’s degree (%)	5401 (66.3)	2146 (85.1)	1948 (73.0)	875 (60.0)	226 (31.6)	206 (26.4)	<.001
Income-to-needs ratio (mean [SD])	4.34 (3.31)	6.05 (3.42)	4.48 (3.12)	3.61 (2.72)	2.22 (2.33)	1.59 (1.58)	<.001
Family history of psychosis (%)	668 (8.2)	179 (7.1)	219 (8.2)	99 (6.8)	76 (10.6)	95 (12.2)	<.001
**B. Area-level deprivation index – median (IQR)**							
% Single parent households	13.51 [8.62, 21.46]	8.11 [5.40, 11.19]	14.40 [10.29, 19.54]	15.12 [11.56, 20.49]	26.90 [20.39, 33.75]	35.66 [28.41, 45.06]	<.001
% Home Ownership	71.91 [54.68, 83.94]	85.80 [78.11, 91.48]	65.58 [53.22, 75.74]	75.89 [64.63, 82.95]	37.56 [22.54, 52.82]	45.75 [35.09, 56.48]	<.001
% Less than 9 years education	2.19 [0.92, 5.06]	0.97 [0.44, 1.85]	2.03 [0.93, 3.78]	3.38 [1.83, 6.06]	16.46 [12.30, 24.24]	5.55 [3.11, 8.28]	<.001
% High school education	92.89 [86.29, 96.40]	96.76 [94.72, 97.97]	93.14 [89.95, 95.96]	89.32 [84.22, 93.37]	69.07 [57.47, 76.91]	81.90 [75.10, 86.74]	<.001
% White collar occupation	94.50 [91.68, 96.63]	95.06 [92.76, 96.95]	95.30 [93.15, 97.08]	91.86 [88.95, 94.75]	91.23 [88.05, 94.06]	94.97 [92.48, 97.37]	<.001
Median family income	75,065.00 [54,375.00, 98,690.00]	105,386.00 [92,389.00, 125,991.00]	74,476.00 [63,500.00, 87,238.00]	65,302.00 [52,626.00, 75,038.00]	40,592.00 [32,267.00, 49,754.00]	36,131.00 [27,640.00, 43,862.25]	<.001
Income disparity	1.96 [1.23, 2.79]	0.87 [0.31, 1.42]	2.07 [1.64, 2.60]	2.26 [1.75, 2.77]	3.15 [2.54, 3.65]	3.91 [3.46, 4.56]	<.001
Median property value	227,200.00 [154,200.00, 321,800.00]	303,600.00 [248,700.00, 427,700.00]	220,600.00 [171,800.00, 310,400.00]	162,700.00 [124,400.00, 215,900.00]	248,500.00 [168,950.00, 317,200.00]	77,700.00 [59,075.00, 107,000.00]	<.001
Median gross rent	1,056.00 [857.00, 1,332.00]	1,376.00 [1,112.00, 1,685.00]	998.00 [868.00, 1,213.75]	921.00 [763.00, 1,134.00]	1,038.00 [910.00, 1,164.50]	747.00 [656.00, 842.00]	<.001
Median monthly mortgage	1,403.00 [1,083.00, 1,723.00]	1,703.00 [1,482.00, 2,102.00]	1,366.00 [1,151.25, 1,636.00]	1,122.00 [899.00, 1,355.50]	1,354.00 [1,083.50, 1,699.50]	759.00 [623.75, 940.00]	<.001
Crowding	1.53 [0.48, 3.49]	0.67 [0.00, 1.69]	1.42 [0.48, 3.04]	1.85 [0.75, 3.37]	13.48 [8.54, 22.95]	2.36 [0.92, 4.70]	<.001
Unemployment	7.22 [4.89, 10.46]	5.16 [3.66, 6.95]	6.94 [5.15, 9.40]	8.26 [5.49, 10.91]	11.91 [9.68, 15.80]	15.39 [11.47, 20.96]	<.001
% Below poverty line	6.53 [3.07, 13.42]	2.36 [1.16, 4.09]	6.94 [4.40, 10.50]	8.93 [5.80, 13.43]	22.82 [15.82, 31.36]	25.59 [18.90, 34.66]	<.001
% Below 138% poverty line	15.01 [8.90, 26.63]	6.87 [4.47, 9.30]	16.07 [12.26, 21.49]	19.17 [13.82, 26.27]	39.52 [31.63, 49.51]	41.27 [34.41, 51.65]	<.001
% Households with no car	4.60 [2.10, 9.64]	1.80 [0.84, 3.45]	6.10 [3.70, 9.94]	4.01 [2.42, 6.42]	10.97 [6.57, 17.35]	21.14 [14.30, 34.16]	<.001
% Poor plumbing	0.00 [0.00, 0.29]	0.00 [0.00, 0.00]	0.00 [0.00, 0.00]	0.00 [0.00, 0.45]	0.00 [0.00, 0.77]	0.00 [0.00, 0.70]	<.001
**C. Child opportunity index – median (IQR)**							
Industrial pollutants	0.40 [−0.45, 0.71]	0.30 [−0.37, 0.64]	0.56 [0.21, 0.85]	−0.76 [−1.54, 0.27]	−0.10 [−0.79, 0.57]	0.69 [0.54, 1.00]	<.001
Hazardous waste dump sites	0.27 [0.27, 0.27]	0.27 [0.27, 0.27]	0.27 [0.27, 0.27]	0.27 [0.27, 0.27]	0.27 [0.27, 0.27]	0.27 [0.27, 0.27]	<.001
Access to food	0.38 [−0.16, 0.67]	0.57 [0.37, 0.73]	0.30 [−0.19, 0.62]	0.23 [−0.16, 0.50]	0.58 [−0.10, 0.85]	−1.97 [−3.28, −0.54]	<.001
Access to green space	−0.30 [−0.90, 0.55]	0.35 [−0.26, 0.85]	−0.70 [−1.07, −0.31]	0.79 [0.17, 1.09]	−1.51 [−1.98, −1.03]	−0.76 [−1.32, −0.35]	<.001
Walkability	10.50 [7.17, 14.00]	7.83 [6.17, 11.00]	13.50 [10.17, 15.33]	7.00 [5.17, 9.33]	14.00 [12.50, 15.50]	12.17 [9.17, 14.83]	<.001
**D. Crime – median (IQR)**							
Total crime	22,761.33 [7,832.00, 53,399.67]	19,950.33 [8,163.00, 36,419.00]	32,088.33 [19,950.33, 57,095.67]	1,304.00 [0.00, 4,445.00]	107,316.00 [36,419.00, 348,049.34]	44,088.00 [22,761.33, 45,073.00]	<.001
**E. Environmental quality – median (IQR)**							
PM2.5 (μg/m3)	7.68 [6.56, 8.60]	7.18 [6.12, 8.17]	7.73 [6.65, 8.54]	7.39 [6.50, 8.18]	8.85 [8.28, 9.74]	8.59 [7.79, 9.40]	<.001
NO2 (ppb)	18.74 [14.75, 22.19]	17.26 [13.73, 21.80]	21.03 [18.42, 25.02]	13.05 [9.92, 15.72]	20.61 [18.48, 22.93]	19.79 [16.36, 21.90]	<.001
O3 (ppb)	40.43 [38.17, 45.13]	40.24 [37.76, 44.18]	40.37 [38.18, 44.70]	42.42 [38.47, 47.37]	42.92 [39.41, 45.91]	39.55 [38.12, 40.78]	<.001
Lead risk	15.73 [6.82, 31.35]	8.60 [4.30, 17.87]	23.79 [10.03, 38.62]	9.14 [5.75, 17.41]	24.43 [13.71, 35.02]	39.16 [26.17, 49.10]	<.001
Proximity to roadways	855.45 [390.82, 1,575.26]	1,161.24 [585.52, 2,093.60]	646.44 [320.07, 1,163.09]	1,079.37 [492.06, 2,216.98]	682.77 [316.00, 1,224.08]	542.44 [249.62, 1,042.95]	<.001
**F. Social vulnerability index – median (IQR)**							
% Ethnoracial minority	46.48 [28.45, 70.43]	33.19 [19.53, 48.13]	45.29 [32.32, 60.89]	43.77 [21.91, 71.31]	89.46 [80.24, 94.36]	80.11 [66.42, 90.96]	<.001
% Non-English speakers	48.57 [27.67, 71.35]	38.89 [23.81, 56.53]	52.86 [33.95, 69.20]	43.26 [20.09, 70.29]	93.25 [89.33, 96.61]	49.35 [23.81, 74.42]	<.001
**G. Urbanicity features – median (IQR)**							
Population density	1,627.60 [786.47, 2,670.60]	1,018.01 [461.56, 1,723.28]	2,081.60 [1,414.74, 2,841.83]	676.25 [148.87, 1,476.91]	4,616.00 [2,991.26, 6,677.55]	2,140.59 [1,434.35, 3,164.03]	<.001
**H. Physical activities – median (IQR])**							
Total physical activities (hours/week)	2.37 [0.87, 4.50]	3.17 [1.58, 5.33]	2.33 [1.00, 4.17]	2.25 [0.67, 4.21]	1.25 [0.00, 3.50]	1.17 [0.00, 3.35]	<.001
Team sports (hours/week)	0.75 [0.00, 2.17]	1.08 [0.00, 2.75]	0.75 [0.00, 2.00]	0.67 [0.00, 2.00]	0.00 [0.00, 1.56]	0.00 [0.00, 1.50]	<.001
Individual sports (hours/week)	0.92 [0.00, 2.29]	1.33 [0.25, 2.79]	1.00 [0.00, 2.25]	0.83 [0.00, 2.25]	0.21 [0.00, 1.50]	0.00 [0.00, 1.50]	<.001
**I. Outcome variable**							
Persistently distressing PLE (%)	1,605 (19.7)	358 (14.2)	469 (17.6)	320 (21.9)	215 (30.1)	243 (31.2)	<.001

*Note:* SOM, Self-organizing map; INR, income-to-needs ratio; PLE, psychotic-like experiences.

a Detailed descriptions of the exposure components and years measured were summarized in Supplementary eTable 2 in the Supplementary Materials.

b P values correspond to the Kruskal–Wallis Rank Sum Test comparing medians, Fisher’s Exact test for proportions, and ANOVA comparing between means.

c Others included participants who identified as American Indian, Alaska Native, Native Hawaiian or Other Pacific Islander, Middle Eastern or North African, reported race not included in the list, did not know their race, did not disclose, or reported multiple races.

### Persistent distressing psychotic-like experiences (PLE)

Participants completed the Prodromal Questionnaire-Brief Child Version (PQBC) to assess PLE (e.g. hallucinations, unusual thought content, paranoia, and other perceptual or thought disturbances) over the past month (Loewy, Pearson, Vinogradov, Bearden, & Cannon, 2011) (see Supplementary eMethods in supplement for the 21 questions assessing PLE). Initially, participants were categorized as either having experienced distressing PLE (rating of at least one PLE > = 3 on a five-point distress scale) or not having experienced distress, following criteria based on prior literature on distressing PLE (Karcher et al., 2022). Participants with distressing PLEs were grouped into persistent and non-persistent categories, with “persistent” indicating distress at two or more follow-ups out of 4 waves (baseline, 1-year, 2-year, and 3-year). This binary categorization was based on prior literature demonstrating such persistency (as opposed to transiency) was specifically associated with a polygenic risk score for schizophrenia (B. S. Ku, Q. Yuan, et al., 2024), greater cognitive deficits, worse social functioning, and more health service utilization (Karcher et al., 2022) as well as a greater risk for future diagnosis of schizophrenia (Dominguez, Wichers, Lieb, Wittchen, & van Os, 2011). Because psychotic symptoms may develop throughout adolescence, we conducted a sensitivity analysis and redefined persistent distressing PLE to incorporate individuals who reported distressing PLE only at the last follow-up.

### Physical activities

Physical activities were derived from the Sports and Activities Involvement Questionnaire (SAIQ). Physical activities were further subcategorized into the team and individual sports (Supplementary eTable 3). The study focused on the level of involvement in 23 physical activities, with caregivers reporting (1) the time spent per session in minutes, (2) the number of days per week of participation, and (3) the number of months per year of engagement. The mean participation hours per week for each sport endorsed in the past 12 months were calculated using the formula: average hours per week per sport in the past year = (time spent × days per week × months per year) / 12/60. (Palmer et al., 2021).

### Statistical analysis

The self-organizing map (SOM) is an unsupervised machine learning technique for multi-dimensional data reduction and visualization, and identifying profiles of correlated characteristics (e.g. pollution concentrations and socioeconomic deprivation). SOM aims to map complex, multi-dimensional data into a simpler, easy-to-visualize format where multiple exposures are grouped into an exposure profile. SOM was used to cluster 29 area-level exposure profiles in this study. See Table 1 and Supplementary eTable 2 for a complete list of exposure variables. The optimal number of profiles was determined using within-cluster sum of squares, between-cluster sum of square statistics, and visual inspection of the exposure profile star plot (Christensen et al., 2022). These methods seek to maximize homogeneity within profiles and heterogeneity between profiles. The reference profile had the highest socioeconomic status and lowest hazardous environmental exposure (e.g. air pollution). Once participants were assigned to an exposure profile, unadjusted and adjusted logistic mixed models were used to estimate the association between exposure profiles, modeled as a categorical exposure, and persistent distressing PLE as well as physical activities (i.e. team and individual sports). The model adjusted for age, sex, family history of psychosis, race and ethnicity, parental education, and income-to-needs ratio, as individual-level fixed effects, and family groups and recruiting sites as random effects.

We then assessed whether team and individual sports would mediate the association between exposure profile and persistent distressing PLE. Mediation analysis was only conducted for exposure profiles that were statistically significantly associated with persistent distressing PLE by modeling each significant exposure profile as a dichotomous exposure variable in the mediation analysis. Detailed definitions and methodology for the mediation analyses were provided in the Supplementary eMethods.

Subsequently, we used another method, weighted quantile sum (WQS) regression, to understand the overall effect of the exposure mixture and how much of the total mixture effect on the outcome is explained by each area-level characteristic within the mixture. WQS is a method that estimates the effect on the outcome of increasing all exposures simultaneously by one quantile, as well as the weighted contribution of each exposure variable to the association between the overall exposure mixture and the outcome while accounting for the complex correlation structure among exposures (Carrico, Gennings, Wheeler, & Factor-Litvak, 2015; Renzetti, Gennings, & Calza, 2023). The detailed methodology of the WQS is summarized in Supplementary eMethods. All SOM analyses were performed using code within the ECM package (https://github.com/johnlpearce/ECM). The other analyses used the following R packages, lme4 (Bates, Mächler, Bolker, & Walker, 2015), lmerTest (Kuznetsova, Brockhoff, & Christensen, 2017), mediation (Tingley, Yamamoto, Hirose, Keele, & Imai, 2014), WQS (Renzetti, Curtin, Allan, Bello, & Gennings, 2016). All analyses were conducted using R version 4.2.1. Statistical significance was determined using an alpha level of 0.05.

## Results

### Descriptive statistics

The study included 8,145 participants aged 9 to 10 years at baseline followed until 13 to 14 years, with 3,868 (47.5%) females, 5,566 (68.3%) non-Hispanic White, 956 (11.7%) non-Hispanic Black, 159 (2.0%) non-Hispanic Asian, and 1,480 (18.4%) Hispanic participants. Among the participants, 5,401 (66.3%) had parents with at least a bachelor’s degree and 668 (8.2%) had a family history of psychosis. Across the four years, 1605 (19.7%) participants had persistent distressing PLE (Table 1). Overall, 1157 (14.21%) of participants did not engage in any physical activity, 2983 (36.62%) and 2559 (31.42%) did not report participating in team sports and individual sports respectively. Individual neighborhood-level characteristics were correlated with each other in the expected direction (Supplementary eFigure 2).

### Five distinct exposure profiles

**Figure 1.**
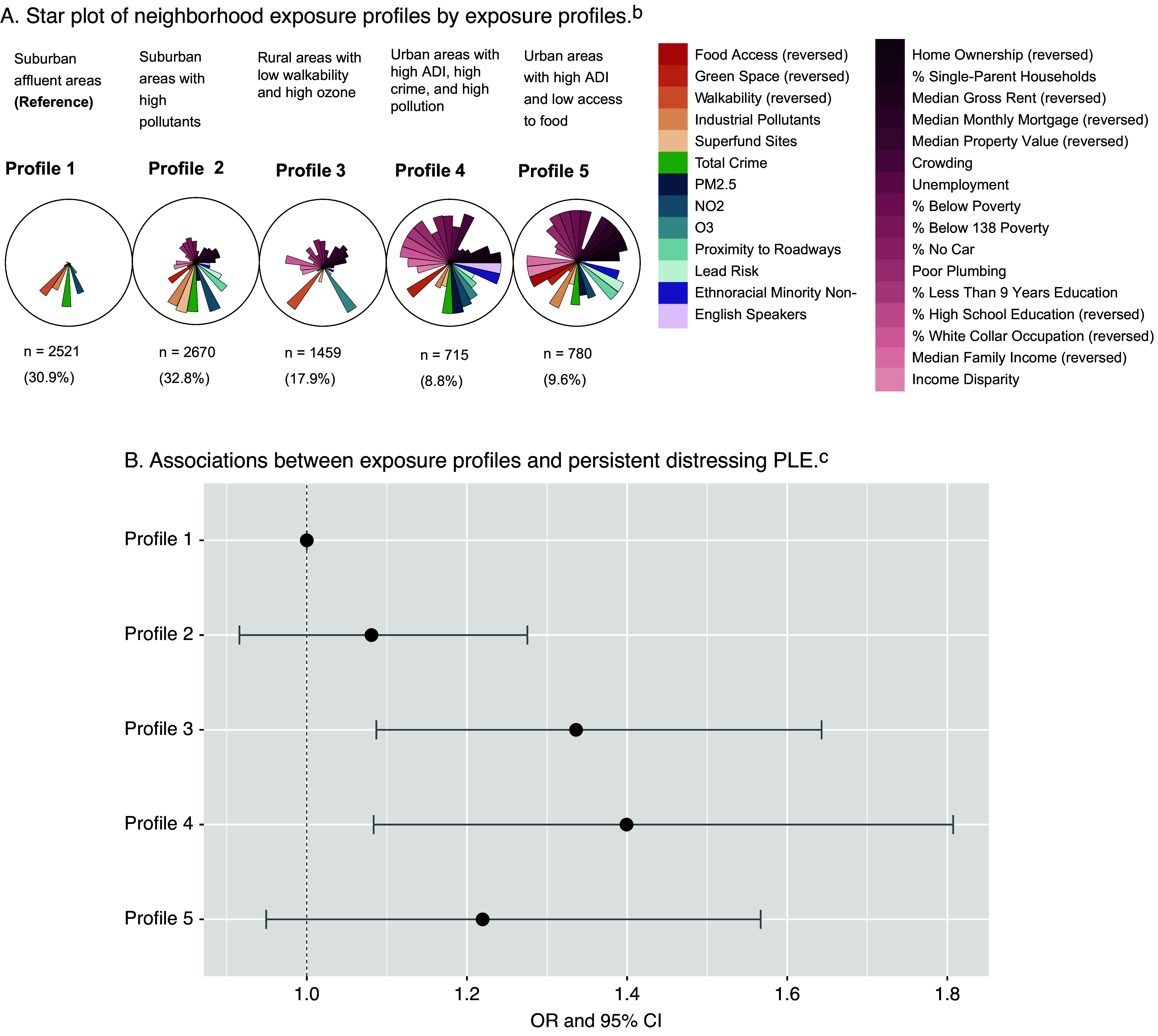
Associations between exposure profiles and persistent distressing PLE.^a^ ^a^Slices from the exposure profiles represent median values of a mixture component. Each circle represents an exposure profile. Profile 1: Suburban affluent areas (n = 2521, 30.9%), Profile 2: Suburban areas with high pollution concentrations (n = 2670, 32.8%), Profile 3: Rural areas with low walkability and high ozone (n = 1459, 17.9%), Profile 4: Urban areas with high ADI, crime, and pollution (n = 715, 8.8%), and Profile 5: Urban areas with high ADI and low access to food (n = 780, 9.6%). ^b^Pink gradient slices correspond to Area-level Deprivation Index (ADI) variables, orange gradient slices correspond to Child Opportunity Index (COI) variables, green slices correspond to total crime, blue gradient slices correspond to environmental exposures, and purple gradient slices correspond to ethnoracial minority and non-English speaker concentration. All variables were coded such that higher scores indicate worse outcomes. ^c^Adjusted logistic regression model results of associations between exposure profiles and persistent distressing PLE, adjusted for age, sex, race and ethnicity, family history of psychosis, parental education, income-to-needs ratio, and included family and sites as random effects.

Five exposure profiles were identified upon visual inspection. Each participant was assigned to one of the five profiles, and their demographics stratified by the exposure profiles are shown in Table 1. Figure 1a presents a detailed description of each profile.

Profile 1 (n = 2521, 30.9%), the reference profile, was characterized as suburban affluent areas with the lowest socioeconomic deprivation, highest education attainment, and low pollution concentrations. Profile 2 (n = 2670, 32.8%) captured suburban areas with higher pollution concentrations, socioeconomic deprivation, and high crime rates compared to Profile 1. Profile 3 (n = 1459, 17.9%) were rural areas with low walkability, high ozone concentrations, and greater socioeconomic disadvantage but high greenspace and low industrial pollution levels. Profile 4 (n = 715, 8.8%) captured urban areas with high ADI, high crime rates, pollution concentrations, crowding, and minimal green space and homeownership. Profile 5 (n = 780, 9.6%) reflects urban areas with high ADI, low food access, income disparity, and high levels of industrial pollutants, alongside the lowest property values and incomes.

### Associations between exposure profiles and persistent distressing PLE

Profile 3, characterized by rural areas with low walkability and high ozone, and Profile 4, characterized by urban areas with high ADI, crime, and pollution, were significantly associated with persistent distressing PLE compared to the reference Profile 1 (Profile 3: adjusted odds ratio (OR): 1.34, 95% CI: 1.09—1.64, p = .006; Profile 4: adjusted OR: 1.40, 95% CI: 1.08—1.81, p = .01) (Table 2, Figure 1b). Sensitivity analyses of (1) an alternative version of persistent distressing PLE and (2) only including participants who lived in their addresses for more than one year showed consistent results (Supplementary eTable 4). Variance inflation factors (VIF) for exposure profiles and individual-level covariates ruled out multicollinearity in our models (Supplementary eTable 5).

**Table 2.** Associations between exposure profiles and persistent distressing PLE^a^

	Features/descriptions	Unadjusted			Adjusted		
		OR	95% CI	*P*	OR	95% CI	*P*
**Exposure**							
Profile 1	Suburban affluent areas (reference)						
Profile 2	Suburban areas with high pollution concentrations	1.33	1.14—1.55	< .001	1.08	0.92—0.28	0.358
Profile 3	Rural areas with low walkability and high ozone	1.69	1.41—2.15	< .001	1.34	1.09—1.64	0.006
Profile 4	Urban areas with high ADI, crime, and pollution	2.42	2.06—2.95	< .001	1.40	1.08—1.81	0.010
Profile 5	Urban areas with high ADI and low access to food	2.67	2.23—3.15	< .001	1.22	0.95—1.57	0.120

*Note:* ADI, area-level deprivation; and PLE, psychotic-like experiences.

a All models included recruiting sites and family groups as two random intercepts. The adjusted model adjusted for individual-level covariates: age, sex, race and ethnicity, family history of psychosis, parents with a bachelor’s degree, and income-to-needs ratio.

### Mediation of the relationship between Profile 3 and persistent distressing PLE by team sports

**Figure 2.**
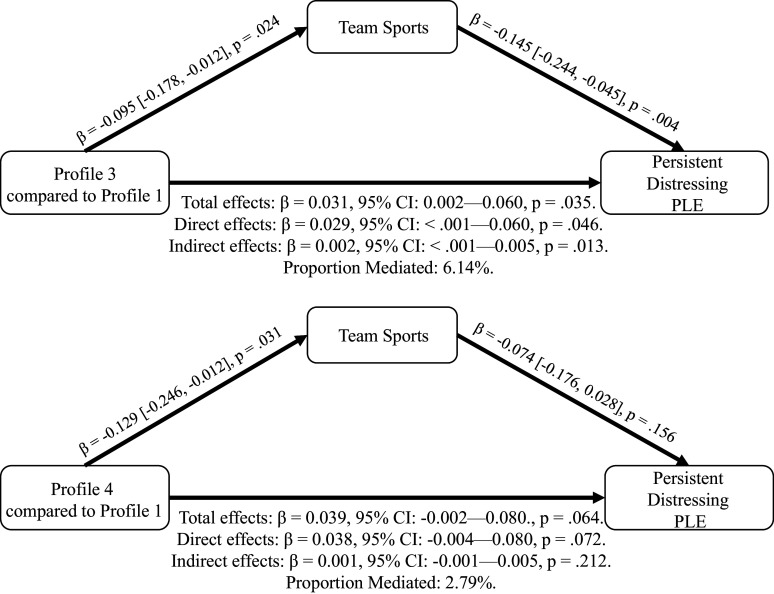
Mediation of the relationship between Profile 3 and persistent distressing PLE by team sports.^a^ ^a^Mediation analysis was conducted for a subgroup of Profile 3 versus Profile 1 (n = 3980), and Profile 4 versus Profile 1 (n = 3236). The mediation analyses included age, sex, race, family history of psychosis, parents with a bachelor’s degree, and income-to-needs ratio as fixed effects and sites as random effects. The coefficients, confidence intervals, and p values of the indirect effects were ascertained by 5000 bootstraps using the mediate() function in the R mediation package (Tingley et al., 2014).

Team sports explained 6.14% of the relationship between Profile 3 and persistent distressing PLE (indirect effects adjusted β: 0.002, 95% CI: <.001—.005, p = .013) (Figure 2). However, team sports did not significantly mediate the association between Profile 4 and persistent distressing PLE (Figure 2). Total physical activities or individual sports did not significantly mediate the association between Profile 3 or Profile 4 and persistent distressing PLE (Supplementary eTables 6 and 7).

### Individual exposure contribution assessed by WQS regression weights

**Figure 3.**
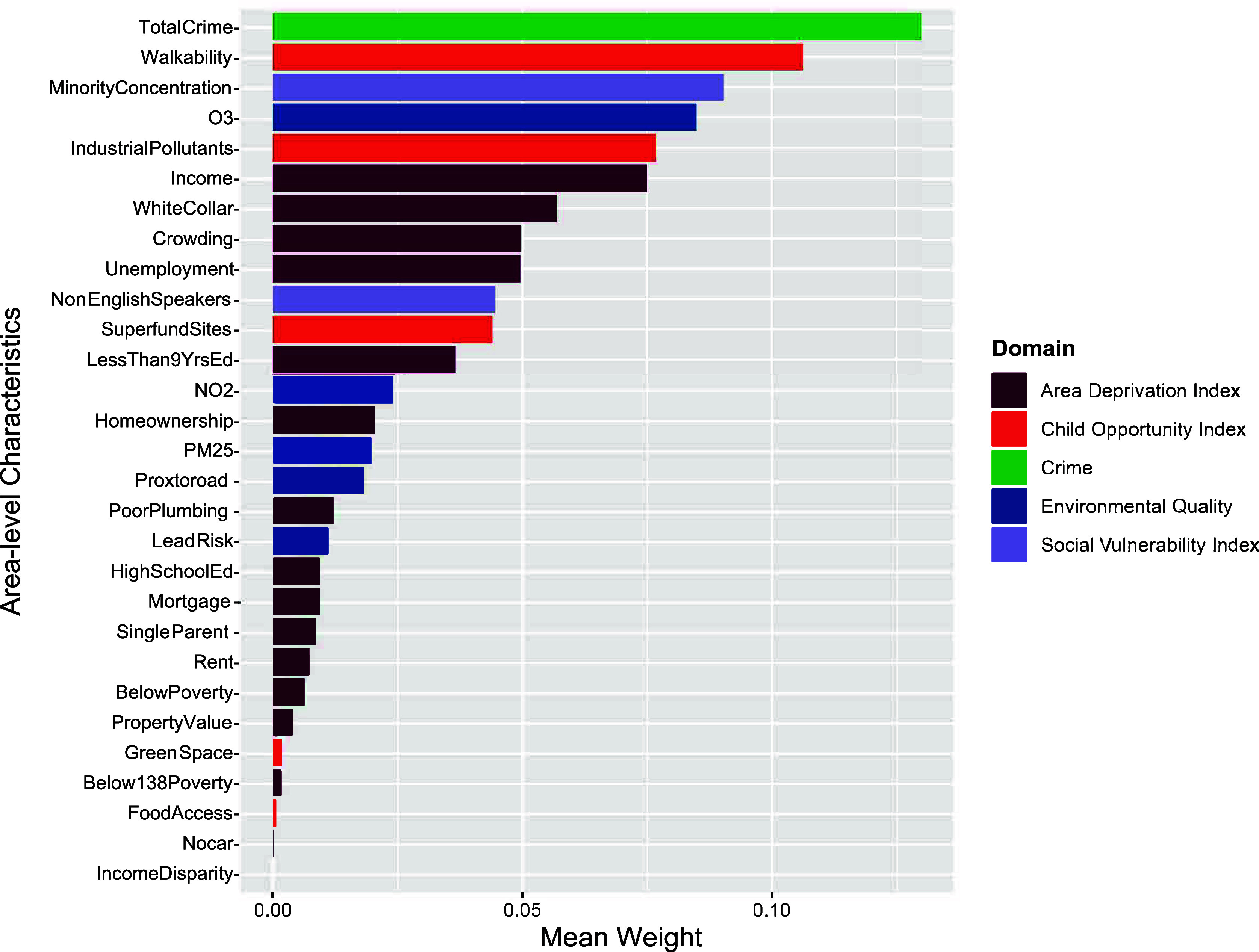
Positive Constrained Weighted Quantile Sum (WQS) Regression Weights.^a^ ^a^The positively constrained model demonstrated that the overall effect of increasing all exposures simultaneously by one decile corresponded to 12% greater odds for endorsement of persistent distressing PLE (adjusted OR: 1.12, 95% CI: 1.02—1.23, p = .03). This tree plot demonstrates the contribution of each exposure to a higher odds of persistent distressing PLE. Mean weights represent the relative contribution of each mixture component to the overall mixture effect for persistent distressing PLE. A list of full names for mixture components and mean weights are shown in Supplementary eTable 8. Covariates included age, sex, family history of psychosis, race, and ethnicity, parents with at least a bachelor’s degree, and income-to-needs ratio.

The positively constrained model demonstrated that the overall effect of increasing all exposures simultaneously by one decile corresponded to 12% greater odds for endorsement of persistent distressing PLE (adjusted OR: 1.12, 95% CI: 1.02—1.23, p = .03). The tree plot presented in Figure 3 demonstrates the contribution of each exposure to a higher odds of persistent distressing PLE with greater total crime having the highest mean weight at 0.130, followed by lower walkability at 0.106, greater ethnoracial minority concentration at 0.090, higher ozone at 0.085, and greater industrial pollution levels at 0.077 (Supplementary eTable 8). The negatively constrained model was not significant (adjusted estimate: < .01, 95% CI: −0.10—0.09, p = .95) (Supplementary eTable 9).

## Discussion

In this study, we identified five unique exposure profiles and investigated their associations with persistent distressing PLE and physical activities. We found two exposure profiles to be associated with greater odds of developing persistent distressing PLE. Rural areas that were less walkable with high ozone (Profile 3) and urban areas with high socioeconomic disadvantage, crime, and pollution (Profile 4) were both uniquely associated with greater odds for persistent distressing PLE even after adjusting for individual-level covariates. It is likely that a combination of factors, including financial stress, food insecurity, limited opportunities for physical activities, and the high concentration of pollution, might heighten the likelihood of PLE (Newbury et al., 2024). Chronic stress has been shown to disrupt the hypothalamic–pituitary–adrenal axis and elevate cortisol levels, which has been associated with neuroanatomical changes linked to psychotic illnesses (Katrina Aberizk et al., 2023; K. Aberizk et al., 2022; Ku et al., 2023).

Subsequently, our mediation analyses suggested that there may be distinct biopsychosocial pathways through which the environment may play a role in the development of psychosis. For example, the degree of involvement in team sports (but not individual sports) modestly mediated the association between Profile 3 and PLE by more than 6%. However, none of the subcomponents of physical activities mediate such relationships for Profile 4. Although the association between Profile 3 and PLE was mainly driven by other factors rather than team sports, it is possible that involvement in team sports (e.g. pick-up basketball) and perhaps other informal social interactions may be less likely to occur in more rural and less walkable places (Aznar et al., 2024). This lack of social engagement with peers may play a role in the future risk of psychotic experiences (Glover, Todd, & Moyer, 2022).

Our findings demonstrating that the association between Profile 4 and PLE was not explained by physical activity suggest that there may be an alternative mechanism through which urban areas characterized by high crime and pollution may be associated with PLE. It is possible that threat from exposure to crime (Bhavsar, Boydell, Murray, & Power, 2014), air pollution (Newbury et al., 2019), and/or financial stress (St-Hilaire, Brunila, & Wachsmuth, 2024) may play a more important role for this subgroup of youth with these specific combinations of exposures. In fact, recent literature has suggested that deprivation and threat may be two environmental factors that underlie partially distinct biological pathways in the development of psychosis (Thomas et al., 2023). Our findings may partially reflect this dimensional model of adversity in psychosis, and it is possible that different environmental factors may impact psychosis through distinct mechanisms.

In addition to the findings using SOM, the WQS regression model also points to similar factors that may drive the development of PLEs. High crime rates and low walkability carried the greatest weight in driving the effects of persistent distressing PLE, which align with high crime rates in Profile 4 and low walkability in Profile 3. Prior research has shown that living in high-crime neighborhoods was associated with subclinical psychotic symptoms, including suspiciousness and paranoia among help-seeking adolescents (Vargas et al., 2020), as well as a higher incidence of first-onset schizophrenia (Bhavsar et al., 2014). Although walkability has not been widely studied in psychosis, places with less walkability tend to have less access to community services and recreational centers (Wang et al., 2023). Access to these resources may have downstream effects on exercise and involvement with team sports, which has been shown to be inversely associated with psychopathology and PLE (Hoffmann et al., 2022).

One of the key strengths of this study is our approach to identifying multidimensional exposure profiles as measured by area-level characteristics, as opposed to traditional single-indexing methods. In addition, we used another exposure mixture method (i.e. WQS) to enhance the rigor of our findings and demonstrated consistent factors that may be driving the association with PLE. Future prospective studies should further investigate the biopsychosocial mechanisms through which neighborhood characteristics, including crime and walkability (and so forth types of crime, park access, street connectivity, and mixed land use) (Leyden, Hogan, D’Arcy, Bunting, & Bierema, 2024) may drive the development of psychotic disorders.

## Limitation

This study has several limitations. First, we excluded several participants due to missing data. Participants excluded were from lower SES households, which may impact the findings’ generalizability. Second, PLEs were self-reported, and physical activities were parent-reported, which may be biased. Third, the outcome of this study is sub-threshold PLEs instead of clinical diagnoses of mental health disorders. Future studies should validate our findings using longitudinal clinical diagnosis data from ABCD follow-up assessments or data from different cohorts. Lastly, this study did not test whether there was a sensitive period in which environmental factors may have a more pronounced effect on long-term psychosis risk. Prospective studies should collect this data and analyze whether environmental factors at various developmental periods may differentially impact psychosis risk.

## Conclusion

This study identified five exposure profiles, of which two were associated with persistent distressing psychotic-like experiences among children and adolescents across four years. These two exposure profiles were characterized by (1) rural areas with low walkability with high ozone (Profile 3), and (2) urban areas with high socioeconomic deprivation and high levels of pollution concentrations (Profile 4). Moreover, we found that less involvement with team sports partially mediated the positive association between Profile 3 (but not Profile 4) and greater odds of developing persistent distressing psychotic-like experiences. These findings suggest that there may be distinct mechanisms through which environmental factors may impact psychosis. Future studies should further explore the mechanisms through which living in certain neighborhoods may confer risk for the development of psychosis.

## Supporting information

Ku et al. supplementary material Ku et al. supplementary material

## Data Availability

The ABCD study anonymized data are released annually and are publicly available via the NIMH Data Archive (NHA). All data from the Adolescent Brain Cognitive Development (ABCD) Study (https://nda.nih.gov/abcd/request-access) are made available to researchers from universities and other institutions with research inquiries following institutional review board and National Institute of Mental Health Data Archive approval.

## Supplementary material

The supplementary material for this article can be found at http://doi.org/10.1017/S0033291725000224.
